# Supplementary material for: HIVprotI: an integrated web based platform for prediction and design of HIV proteins inhibitors
Source: J Cheminform. 2018 Mar 9;10:12. doi: 10.1186/s13321-018-0266-y (PMC5845081; doi:10.1186/s13321-018-0266-y)
Supplement: Supplementary file 2 — Additional file 2. Source code of HIVProtI web server. [file 13321_2018_266_MOESM2_ESM.zip › HIVprotI_Source-code/hivprotI/weka-3-6-6/doc/weka/gui/sql/event/ConnectionEvent.html]

ConnectionEvent


---


|  |  |  |
| --- | --- | --- |
| **Overview** \| **Package** \| **Class** \| **Tree** \| **Deprecated** \| **Index** \| **Help** \| **Weka's home** | |  |
| PREV CLASS   **NEXT CLASS** | **FRAMES**    **NO FRAMES**     **All Classes** |  |
| SUMMARY: NESTED \| FIELD \| CONSTR \| METHOD | DETAIL: FIELD \| CONSTR \| METHOD |  |


---


## weka.gui.sql.event Class ConnectionEvent

```
java.lang.Object
  java.util.EventObject
      weka.gui.sql.event.ConnectionEvent
```

**All Implemented Interfaces:**: java.io.Serializable

---

``` public class ConnectionEvent extends java.util.EventObject ```

An event that is generated when a connection is established or dropped.

**Version:**
:   $Revision: 1.2 $

**Author:**
:   FracPete (fracpete at waikato dot ac dot nz)

**See Also:**: `ConnectionListener`, Serialized Form

---

| **Field Summary** | |
| --- | --- |
| `static int` | `CONNECT`             it was a connect try |
| `static int` | `DISCONNECT`             it was a disconnect |


**Constructor Summary**

`ConnectionEvent(java.lang.Object source,
int type,
DbUtils utils)`
  
          constructs the event

`ConnectionEvent(java.lang.Object source,
int type,
DbUtils utils,
java.lang.Exception ex)`
  
          constructs the event


| **Method Summary** | |
| --- | --- |
| `boolean` | `failed()`             whether an exception happened and is stored |
| `DbUtils` | `getDbUtils()`             returns the DbUtils instance that is responsible for the connect/disconnect. |
| `java.lang.Exception` | `getException()`             returns the stored exception, if any (can be NULL) |
| `int` | `getType()`             returns the type of this event, CONNECT or DISCONNECT |
| `boolean` | `isConnected()`             returns whether the connection is still open. |
| `java.lang.String` | `toString()`             returns the event in a string representation |

**Methods inherited from class java.util.EventObject**

`getSource`

**Methods inherited from class java.lang.Object**

`equals, getClass, hashCode, notify, notifyAll, wait, wait, wait`

**Field Detail**

### CONNECT

```
public static final int CONNECT
```

:   it was a connect try

    **See Also:**: Constant Field Values

---


### DISCONNECT

```
public static final int DISCONNECT
```

:   it was a disconnect

    **See Also:**: Constant Field Values


**Constructor Detail**

### ConnectionEvent

```
public ConnectionEvent(java.lang.Object source,
                       int type,
                       DbUtils utils)
```

:   constructs the event

    **Parameters:**: `source` - the source that generated this event: `type` - whether CONNECT or DISCONNECT happened: `utils` - the DatabaseUtils isntance responsible for the connection

---


### ConnectionEvent

```
public ConnectionEvent(java.lang.Object source,
                       int type,
                       DbUtils utils,
                       java.lang.Exception ex)
```

:   constructs the event

    **Parameters:**: `source` - the source that generated this event: `type` - whether CONNECT or DISCONNECT happened: `utils` - the DatabaseUtils isntance responsible for the connection: `ex` - a possible exception, if not successful


**Method Detail**

### getType

```
public int getType()
```

:   returns the type of this event, CONNECT or DISCONNECT

    :   **Returns:**: the type of this event **See Also:**: `CONNECT`, `DISCONNECT`

---


### failed

```
public boolean failed()
```

:   whether an exception happened and is stored

    :   **Returns:**: whether an exception happened

---


### isConnected

```
public boolean isConnected()
```

:   returns whether the connection is still open.

    :   **Returns:**: whether the connection is still open

---


### getException

```
public java.lang.Exception getException()
```

:   returns the stored exception, if any (can be NULL)

---


### getDbUtils

```
public DbUtils getDbUtils()
```

:   returns the DbUtils instance that is responsible for the
    connect/disconnect.

    :   **Returns:**: the responsible DbUtils instance

---


### toString

```
public java.lang.String toString()
```

:   returns the event in a string representation

    :   **Overrides:**: `toString` in class `java.util.EventObject`
    :   **Returns:**: the event in a string representation


---


|  |  |  |
| --- | --- | --- |
| **Overview** \| **Package** \| **Class** \| **Tree** \| **Deprecated** \| **Index** \| **Help** \| **Weka's home** | |  |
| PREV CLASS   **NEXT CLASS** | **FRAMES**    **NO FRAMES**     **All Classes** |  |
| SUMMARY: NESTED \| FIELD \| CONSTR \| METHOD | DETAIL: FIELD \| CONSTR \| METHOD |  |


---
